# Probing the Role of the Conserved Arg174 in Formate Dehydrogenase by Chemical Modification and Site-Directed Mutagenesis

**DOI:** 10.3390/molecules26051222

**Published:** 2021-02-25

**Authors:** Mohammed Hamed Alqarni, Ahmed Ibrahim Foudah, Magdy Mohamed Muharram, Haritium Budurian, Nikolaos E. Labrou

**Affiliations:** 1 Department of Pharmacognosy, College of Pharmacy, Prince Sattam Bin Abdulaziz University, Alkharj 11942, Saudi Arabia; a.foudah@psau.edu.sa; 2 Department of Pharmaceutics, College of Pharmacy, Prince Sattam Bin Abdulaziz University, Alkharj 11942, Saudi Arabia; m.moharm@psau.edu.sa; 3 Department of Microbiology, College of Science, Al-Azhar University, Nasr City, Cairo 11884, Egypt; 4 Laboratory of Enzyme Technology, Department of Biotechnology, School of Food, Biotechnology and Development, Agricultural University of Athens, 75 Iera Odos Street, GR-11855 Athens, Greece; haris.bod@gmail.com

**Keywords:** formate dehydrogenase, NAD^+^ binding site, site-directed mutagenesis

## Abstract

The reactive adenosine derivative, adenosine 5′-*O*-[S-(4-hydroxy-2,3-dioxobutyl)]-thiophosphate (AMPS-HDB), contains a dicarbonyl group linked to the purine nucleotide at a position equivalent to the pyrophosphate region of NAD^+^. AMPS-HDB was used as a chemical label towards *Candida boidinii* formate dehydrogenase (*Cb*FDH). AMPS-HDB reacts covalently with *Cb*FDH, leading to complete inactivation of the enzyme activity. The inactivation kinetics of *Cb*FDH fit the Kitz and Wilson model for time-dependent, irreversible inhibition (K_D_ = 0.66 ± 0.15 mM, first order maximum rate constant k_3_ = 0.198 ± 0.06 min^−1^). NAD^+^ and NADH protects *Cb*FDH from inactivation by AMPS-HDB, showing the specificity of the reaction. Molecular modelling studies revealed Arg174 as a candidate residue able to be modified by the dicarbonyl group of AMPS-HDB. Arg174 is a strictly conserved residue among FDHs and is located at the Rossmann fold, the common mononucleotide-binding motif of dehydrogenases. Arg174 was replaced by Asn, using site-directed mutagenesis. The mutant enzyme *Cb*FDHArg174Asn was showed to be resistant to inactivation by AMPS-HDB, confirming that the guanidinium group of Arg174 is the target for AMPS-HDB. The *Cb*FDHArg174Asn mutant enzyme exhibited substantial reduced affinity for NAD^+^ and lower thermostability. The results of the study underline the pivotal and multifunctional role of Arg174 in catalysis, coenzyme binding and structural stability of *Cb*FDH.

## 1. Introduction

NAD^+^-dependent formate dehydrogenase (FDH, EC 1.2.1.2) catalyzes the reversible conversion of formate anion to carbon dioxide and two electrons [1,2,3]. FDHs, based on their structural features and cofactor requirements are classified into two groups [4]: the NAD^+^-independent and the NAD^+^-dependent. NAD^+^-independent FDHs contain at the active site oxygen-labile compounds (e.g., tungsten, molybdenum, iron-sulfur clusters, selenocysteine). They are sensitive enzymes and therefore, NAD^+^-independent FDHs appear to have limited suitability in bicatalysis and other biotechnological applications. On the other hand, NAD^+^-dependent FDHs are stable enzymes and have been exploited in several applications [5,6,7]. For instance, a wide range of FDHs have found successful applications in the development of efficient NAD(H) regeneration systems [6,8,9,10,11], as well as CO_2_-reduction systems [7,12,13,14,15,16].

Reducing the atmospheric CO_2_ level and the biological CO_2_ fixation is of paramount importance to combat global warming and fossil-fuel shortages [17]. The reduction of CO_2_ using FDHs is considered very attractive since it allows the production of valuable chemicals, such as formic acid and methanol [9,15,18]. Other uses of FDHs include application in analytical biochemistry for the determination of formic acid in biological and clinical samples [19,20] and use as antidote against methanol poisoning cases [21,22]. Therefore, the study of the structure and functional properties of FDHs is of both academic and practical interests.

The Rossmann fold (or mononucleotide-binding motif) is one of the most common and widely distributed super-secondary structure of many enzymes that recognize NAD(H), NADP(H) and related cofactors [23,24]. The Rossmann fold is formed by two α-helices and three β-strands in the alternating pattern βαβαβ. The pyrophosphate binding region of the Rossmann fold is positioned at the *N*-terminus of the first α-helix, formed by conserved Gly residues. This glycine-rich phosphate-binding loop contains three Gly residues arranged as GXGXXG, where X: is any amino acid residue [23,24].

The identification of specific binding sites and reactive residues in proteins is a useful strategy in protein biochemistry [25,26,27]. Chemical modification, molecular modelling and affinity labelling are powerful methods for investigating the structural role, the reactivity and chemistry of specific residues [28,29,30,31,32]. Modification of arginine residues using dicarbonyl compounds is a common method to identify functional or reactive arginine residues in proteins [33,34,35]. Several dicarbonyl compounds have been utilized and the most effective ones are phenylglyoxal, 2,3-butanedione and cyclohexanedione. Since these compounds do not provide specificity and are able to react with all the solvent exposed Arg residues, the chemical labelling approach using such dicarbonyl compounds is very challenging [33,34,35,36]. For example, previously published work showed that 2,3-butanedione is able to react with 12 Arg residues in bacterial FDH, although the primary target of 2,3-butanedione is the active-site Arg residue [37].

**Figure 1:**
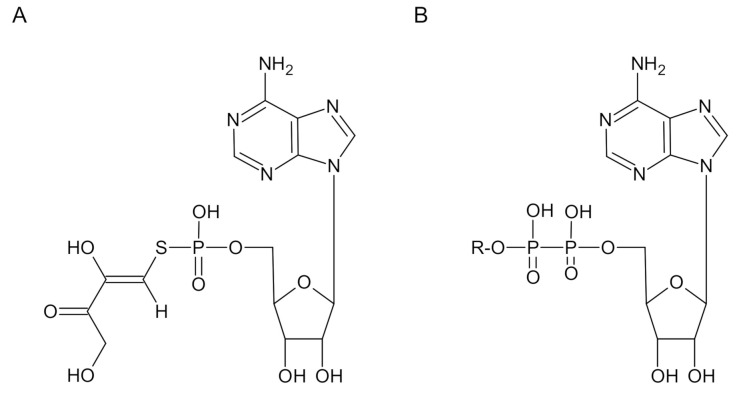
The structure of the the reactive adenosine derivative, adenosine 5′-*O*-[*S*-(4-hydroxy-2,3-dioxobutyl)]-thiophosphate (AMPS-HDB) (**A**). The structure of the ADP part of NAD^+^ is also presented (**B**), for comparison. Where R: nicotinamide ribose part of NAD^+^.

The reactive adenosine derivative adenosine 5′-*O*-[*S*-(4-hydroxy-2,3-dioxobutyl)]-thiophosphate (AMPS-HDB), contains a dicarbonyl group linked to the purine nucleotide at a position such that it can structurally mimics the pyrophosphate region of NAD^+^ (Figure 1). AMPS-HDB is expected to bind to nucleotide binding enzymes and function as a chemical label for nucleotide sites [38]. Similar analogues have been used as affinity labels for other enzymes [32,38].

The FDH from the methylotrophic yeast *Candida boidinii* (*Cb*FDH) has been used as a model formate dehydrogenase in enzymology [1,3,4,5]. Furthermore, detailed studies of *Cb*FDH are justified by the considerable biotechnological potential of this enzyme. For example, protein engineering studies of *Cb*FDH, aiming to alter its coenzyme specificity (NADP^+^ vs NAD^+^) is of practical importance for the development of an efficient enzyme useful in NADP^+^ regeneration systems [5]. To this end, the characterization of coenzyme binding residues can guide rational redesign approaches.

In the present work, chemical modification, site-directed mutagenesis and biocomputing analysis were employed to provide new knowledge on the structure and function of *Cb*FDH. The approach is expected to be useful to extent the range of available methodologies and in complementing the information obtained through X-ray crystallography and other biophysical methods.

## 2. Results and Discussion

### 2.1. Structural Context

**Figure 2:**
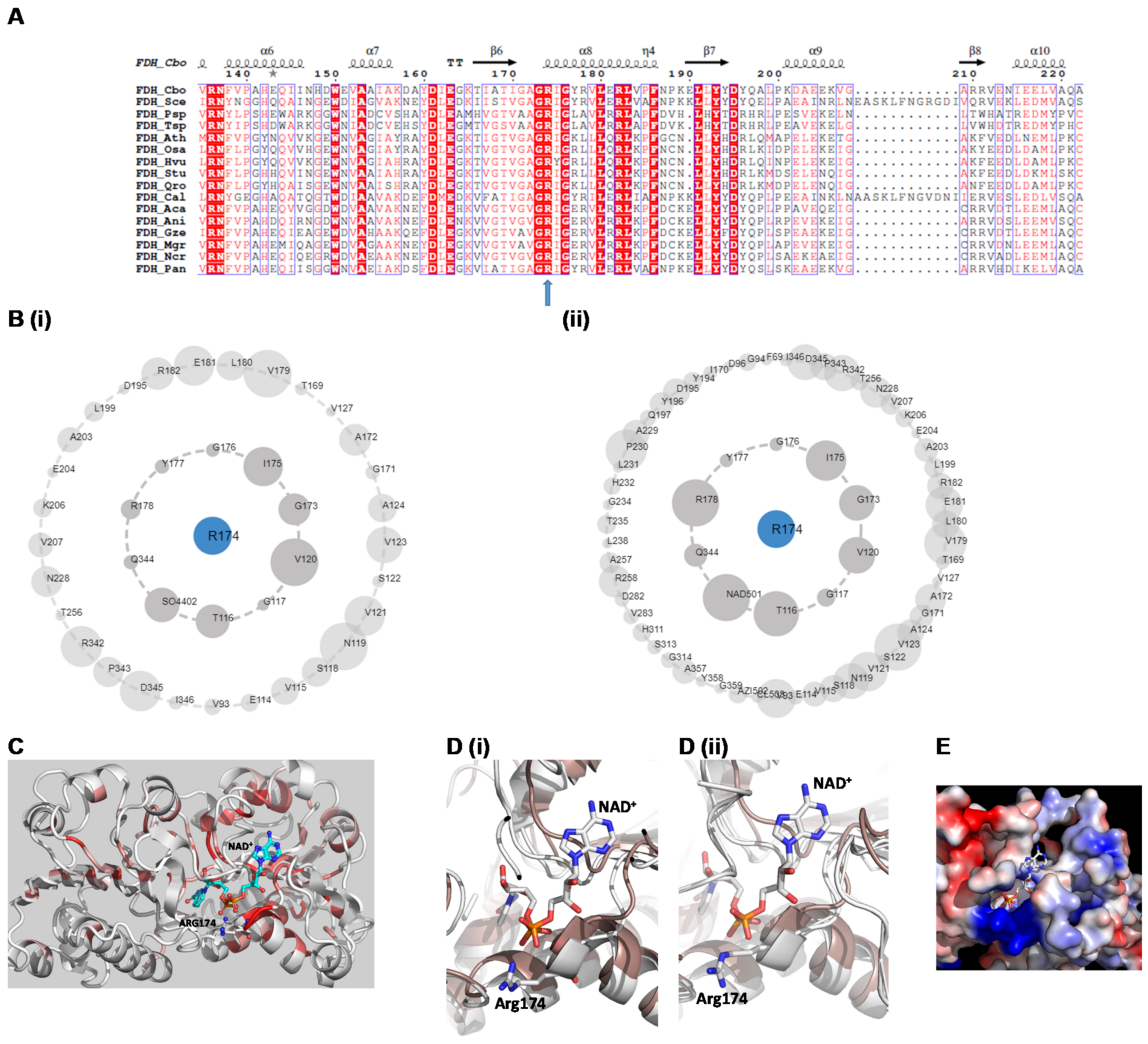
Structural analysis. (**A**) Aminoacid sequence alignments of NAD^+^-binding region of plant, bacteria, fungi and yeasts FDHs. Abbreviations and National Center for Biotechnology Information (NCBI) accession codes are: FDH_Cb, CAA09466; FDH_Sc, NP_015033, FDH_Pan, P33677; FDH_Cal, XP_711169; FDH_Psp, P33160; FDH_Tsp, BAC92737; FDH_Ath, NP_196982; FDH_Stu, Q07511; FDH_Osa, NP_001057666; FDH_Qro, CAE12168; FDH_Hvu, Q9ZRI8; FDH_Aca, AAV67968; FDH_Ani, XP_664129; FDH_Mgr, AAW69358; FDH_Gze, XP_386303; FDH_Ncr, XP_961202. The alignments were created using Clustal O. The secondary structure of *Cb*FDH (PDB code 2FSS) and numbering are shown above the alignment. The arrow below the alignments depicts Arg174. (**B**) Asteroid Plot of interactions of Arg174 in NAD^+^-free (**i**) (Protein Data Bank code: 5dna) and NAD^+^-bound (**ii**) (Protein Data Bank code: 5dn9) structures. The inner ring indicates first shell of immediate atomic contacts. The outer ring indicates second shell of extended atomic contacts. The size of the circle is proportional to the total number of contacts that each residue is involved in with any of the residues in the ring inward to it. The diagrams were created by Protein Contact Atlas [38]. (**C**) Diagram of the subunit of *Cb*FDH with NAD^+^ bound to the active site. (**D**) Movement of Arg174 upon NAD binding. (**i**) The conformation of Arg174 in NAD^+^-bound structure (PDB code: 5dn9, colored grew). (**ii**) The conformation of Arg174 in NAD^+^-free structure (PDB code: 5dna, colored brown). Both structures (5dn9 and 5dna) were superimposed. (**E**) Coulombic surface coloring of the electrostatic potential in the NAD^+^ binding site. NAD^+^ is shown in stick representation and colored according to the atom type.

Analysis of the crystal structure of *Cb*FDH (PDB code 5DN9) revealed the presence of the conserved pyrophosphate-binding site at the Rossmann fold [23,24]. This site forms a loop, composed by three conserved Gly residues arranged in the patterns GXGXXG (Figure 2A). In all FDHs, the first amino acid residue after the second Gly residue is an arginine residue (Arg174 according to *Cb*FDH numbering, Figure 2A). The plausible function of Arg174 is its participation in electrostatic binding and orientation of the pyrophosphate group of the coenzyme to the NAD(H) site. This residue is exposed to the solvent and is accessible for chemical modification, allowing the investigation of its reactivity and contribution to coenzyme binding and catalysis by chemical means. Arg174 (Figure 2B,C) is involved in a wide range of interactions with neighbour residues and establishes direct interaction with the pyrophosphate group of NAD^+^ with distance 2.94 Å. The exact interaction pattern of Arg174 is shown in Figure 2B. Arg174 also undergoes large structural rearrangement upon NAD^+^ binding (Figure 2D). In particular, its side chain is moved 2 Å towards coenzyme, contributing to the induced fit adaptation that is operated by *Cb*FDH [1,6].

The reactive adenosine derivative, AMPS-HDB (Figure 1) possesses a dicarbonyl group at the 5′-position of ribose, equivalent to the pyrophosphate region of NAD^+^ [39]. Since the reactive dicarbonyl group is located adjacent to the 5′-position, AMPS-HDB is expected to react with Arg residues at the pyrophosphate region (e.g., Arg174) of the adenine nucleotide-binding site of *Cb*FDH.

### 2.2. Kinetics of Reaction of AMPS-HDB with CbFDH

**Figure 3:**
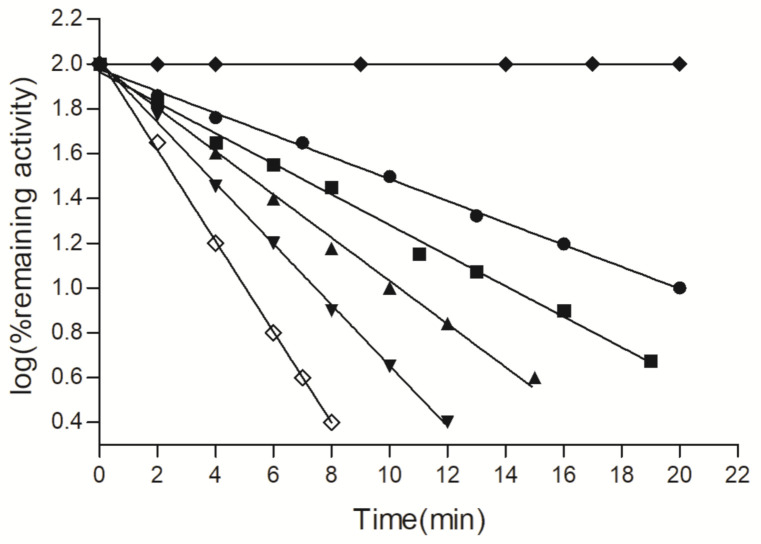
Inactivation of recombinant *Cb*FDH by AMPS-HDB. Enzyme was incubated in the absence (♦) and in the presence of AMPS-HDB: 0.05 mM (•); 0.1 mM (■); 0.2 mM (▲); 0.3 mM (▼); 0.5 mM (◊); at pH 8.0 and 25 °C. At the times indicated, aliquots were withdrawn and assayed for enzymatic activity.

Incubation of *Cb*FDH with 0.3 mM AMPS-HDB at 25 °C and pH 8.0 resulted in time-dependent loss of enzymatic activity (Figure 3). In contrast, the control enzyme, incubated in the absence of AMPS-HDB, under identical conditions, displayed no loss of enzyme activity. The plot of residual activity versus incubation time was linear as shown in Figure 3, indicating a pseudo-first order kinetics with a rate constant 0.059 min^−1^.

**Figure 4:**
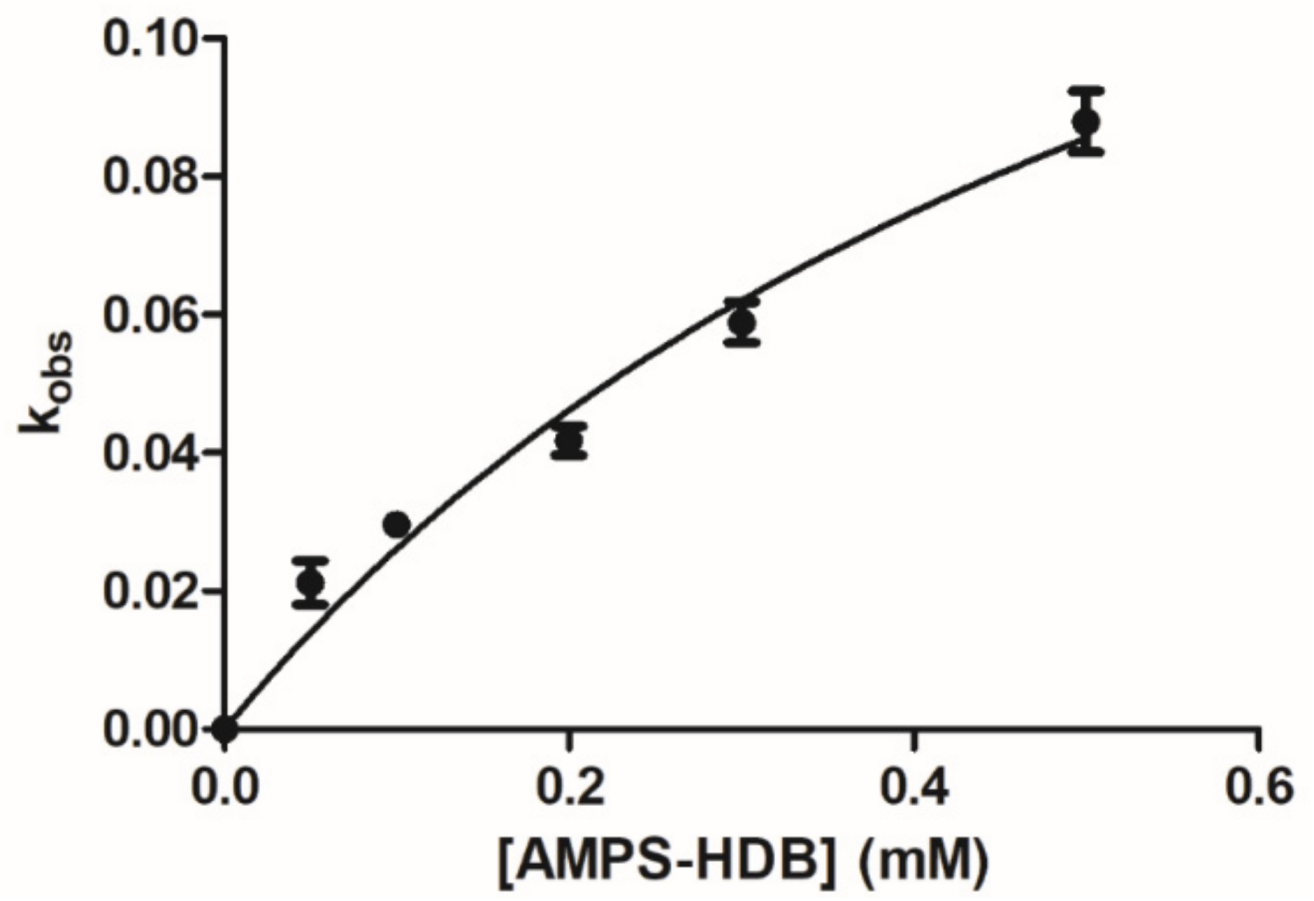
Dependence of the pseudo-first-order rate constant of inactivation (k_obs_) on the concentration of AMPS-HDB. *Cb*FDH was incubated with various concentrations of AMPS-HDB (0–0.5 mM) and the pseudo-first-order rate constants for the inactivation reaction were calculated from the plots as illustrated in Figure 1.

*Cb*FDH was incubated with 0.05–0.5 mM of AMPS-HDB as described above, to determine the rate of inactivation at different AMPS-HDB concentrations. The dependence of the observed rate of inactivation on AMPS-HDB concentration is depicted in Figure 4. The hyperbolic dependence that was found is typical for a reaction that obeys pseudo-first order saturation kinetics [34,40,41]. This is consisted with the formation of a reversible enzyme:AMPS-HDB complex (Michaelis binary complex), prior to covalent modification of the enzyme.

Therefore, the inactivation kinetics of *Cb*FDH fit the Kitz and Wilson model for time-dependent, irreversible inhibition, according to the following Equation (1) [34,40,41,42,43]:

(1) $$E\;+\;AMPS-HDB\;\overset{K_{D}}{↔}E:AMPS-HDB\overset{k_{3}}{\to }E\;-\;AMPS-HDB$$

where: E is the free enzyme; E:AMPS-HDB is the Michaelis binary complex and E-AMPS-HDB is the covalent product. The steady-state rate equation can be described by the following Equation (2) [40]:

(2) $$k_{obs}\;=\;k_{3}\left[AMPS-HDB\right]/\left(K_{D}\;+\;\left[AMPS-HDB\right]\right)$$

where: k_obs_ is the observed rate of inactivation (min^−1^) for each AMPS-HDB concentration; k_3_ is the maximum rate of inactivation (min^−1^), and K_D_ is the apparent dissociation constant of the Michaelis binary complex. A least squares fit of the experimental data yields, K_D_ 0.66 ± 0.15 mM and apparent maximum rate constants (k_3_) 0.198 ± 0.06 min^−1^ (Figure 4).

**Scheme 1:**
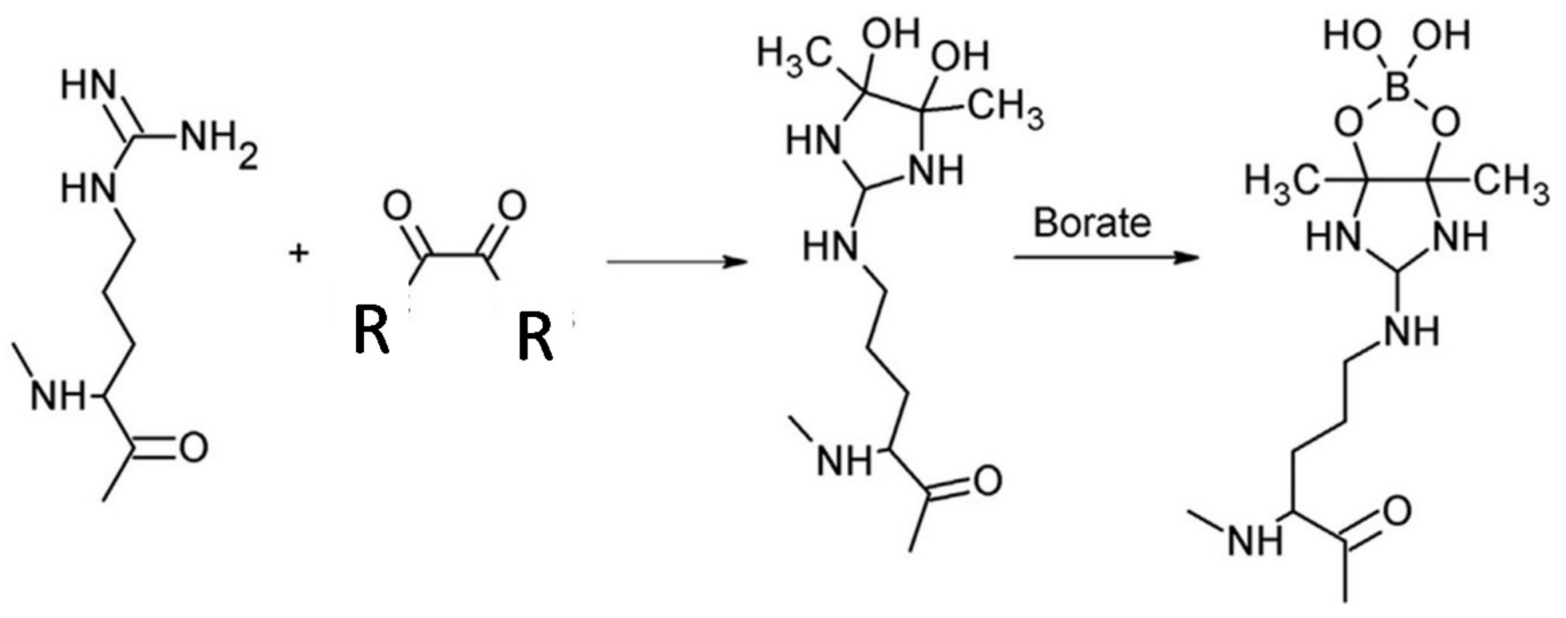
The reaction of an Arg residue with dicarbonyl compound in borate buffer.

Extensive investigations have establish that the mechanism and the reactivity of the guanidino group of Arg residues with dicarbonyl compounds depend on the pK_a_ of the guanidino group [36]. Although the guanidino group of arginine is very basic (pKa 12.5 in free arginine), it is the unprotonated free base that reacts with dicarbonyl compounds [35,36]. The modification of *Cb*FDH by AMPS-HDB presumably proceeds through the pathway utilizing the unprotonated form of Arg174 (Scheme 1). *Cb*FDH was inactivated in the presence of borate ions, borate ions contribute to the formation of a stabilized cyclic borate ester [35,36].

Since the reactivity of individual arginyl side-chains toward dicarbonyl compounds is primarily affected by the pK_a_ value of the guanidinium group, we can assume that Arg174 has a slightly perturbed pK_a_. This is probably due to the strong positive electrostatic potential of the pyrophosphate-binding site as (Figure 2E). The strong positive electrostatic potential can affect the pK_a_ of Arg174, enhancing its reactivity toward the AMPS-HDB. Certainly, the pK_a_ of Arg174 can not be altered significantly to assume that it has lost its positive charge under the conditions employed in the present study.

### 2.3. Effect of Substrates on the Reaction of AMPS-HDB with CbFDH

**Figure 5:**
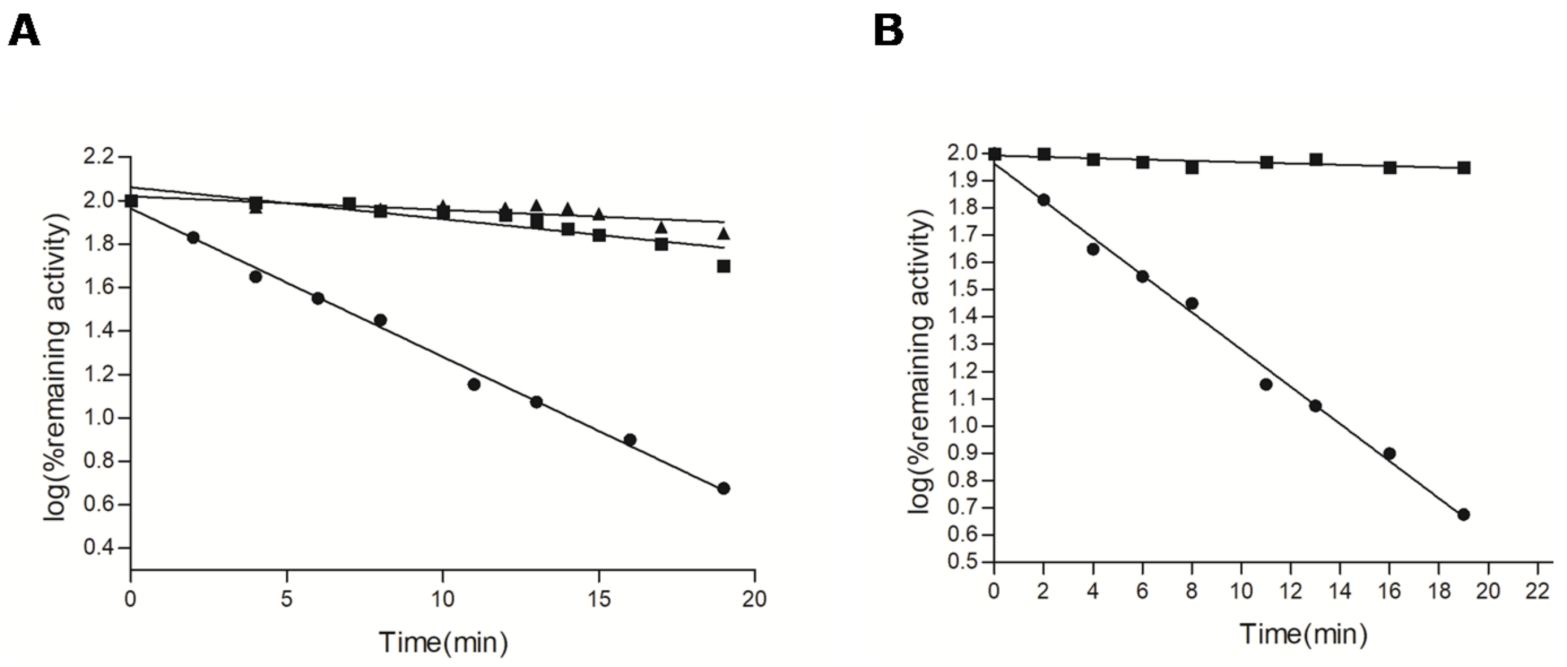
The effects of nucleotides and mutation at position 174 on the inactivation of *Cb*FDH by AMPS-HDB. (A) Time course of inactivation of *Cb*FDH by AMPS-HDB (0.1 mΜ) at pH 8.0 and 25 °C, in the absence (●) or in the presence of NAD^+^ (■, 1 mM) or NADH (▲, 1 mM). (B) Inactivation of the mutant enzyme Arg174Asn by AMPS-HDB (0.1 mΜ) (■). At the times indicated, aliquots were withdrawn and assayed for enzymatic activity.

The specificity of the reaction can be evaluated by the ability of native enzyme’s ligands (e.g., substrate, inhibitor) to influence (promote or inhibit) the rate of inactivation [34,44,45,46,47]. In the present work, the effect of the substrates, formate, NAD^+^ and NADH on the reaction of AMPS-HDB with CoFDH was investigated (Figure 5). In the absence of protecting ligands, *Cb*FDH retained less than 4% activity after incubation with 0.1 mM AMPS-HDB for 20 min. However, in the presence of NAD^+^ and NADH the enzyme retained >80% of its initial activity (Figure 5A). These observations indicate that the nucleotides compete with AMPS-HDB in occupying the target site on *Cb*FDH. In contrast to nucleotides, formate fails to protect *Cb*FDH from inactivation, even at high concentration (50 mM, data not shown). This is probably due to the compulsory ordered Bi-Bi kinetic mechanism that is obeyed by the enzyme [1,6]. According to this mechanism, the enzyme binds formate after the formation of the binary enzyme:NAD^+^ complex. Therefore, enzyme:formate binary complex is not formed under the typical assay conditions.

### 2.4. Site-Directed Mutagenesis of Arg174 Mutant Enzyme

To provide further experimental evidence and establish the involvement of Arg174 in the reaction with AMPS-HDB, site-directed mutagenesis experiments were carried out. Arg174 was mutated to Asn, and the mutant enzyme was subjected to inactivation studies (Figure 5B). Asn is a polar amino acid with smaller side-chain, compared to Arg. The smaller size allows the correct local folding of the protein, avoiding large structural perturbations. We avoided the substitution of Arg174 with Lys, since the ε-amino group of Lys is able to react with carbonyl compounds through the formation of Schiff base.

The results showed that the mutant enzyme was completely resistant to inactivation by AMPS-HDB, compared to the wild-type enzyme. This experiment demonstrates that Arg174 reacts with is AMPS-HDB, as predicted by the preceding labelling and molecular modelling studies. Comparison of the circular dichroism (CD) spectra of native and mutated enzyme indicated the absence of any significant structural perturbation caused by the mutation (see Supplementary Figure S1). This observation rules out the possibility that the resistance to inactivation of the Arg174Asn mutant enzyme was due to conformational changes in the structure of the enzyme.

### 2.5. Kinetics and Stability Analysis of the Wild-Type and Mutant Arg174Asn Enzymes

Kinetic analysis of the wild-type and mutant enzymes was achieved and the results are listed in Table 1. The results showed that the mutant Arg174Asn enzyme displays unchanged K_m_ value for formate (3.1 ± 0.2 mM). On the other hand, the mutant enzyme shows higher K_m_ value for NAD^+^ (1.27 ± 0.1 mM), compared to the wild type enzyme.

The dramatic increase (~32-fold) of K_m_ value for NAD^+^ suggests a role of Arg174 as one of the main structural determinant affecting the affinity of *Cb*FDH for coenzyme. Kinetic inhibition studies indicated that NADH and AMP are competitive inhibitors with respect to NAD^+^. As shown in Table 1, the K_i_ value of each compound is increased significant for the mutant enzyme, suggesting that the affinity of the enzyme for NADH and AMP has been perturbed. In addition, the reduction of k_cat_ observed for the mutant enzyme presumably underline the pivotal role of Arg174 in the productive binding and correct orientation of coenzyme in the enzyme’s active site or in the transition state formation (activation energy).

**Figure 6:**
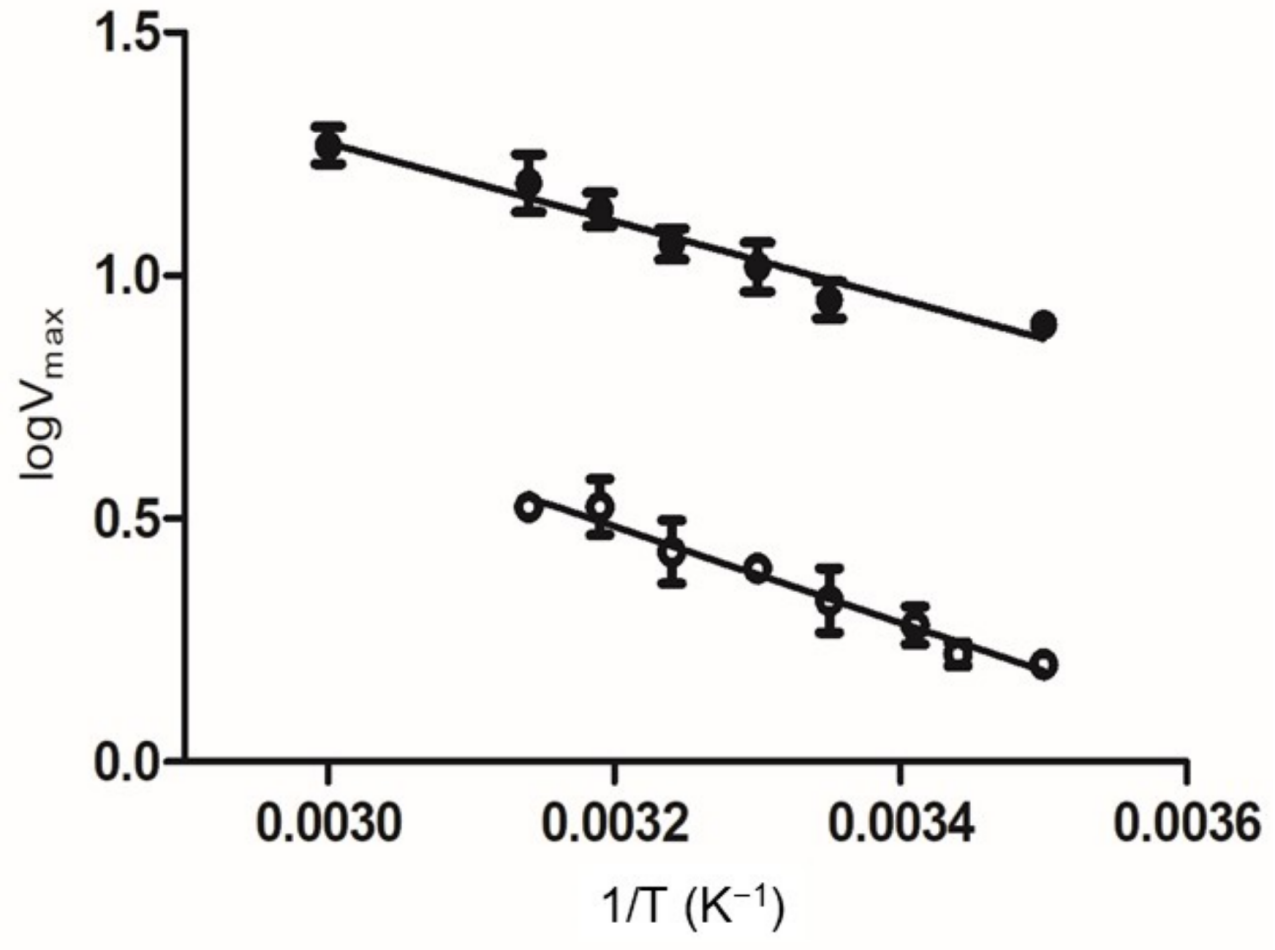
Effect of temperature on V_max_ for the wild-type and the mutant Arg174Asn enzyme. The data were fitted to the Arrhenius equation for the wild-type, (●); and for the mutant (○) enzyme.

To investigate the effect of single-point mutation on the activation energy of the catalytic reaction we studied the effect of temperature on V_max_. The fitting of experimental data to the Arrhenius equation showed a linear dependence for both the wild-type and mutant enzymes (Figure 6). The mutant enzyme exhibits higher activation energy (18.9 ± 1.1 kJ/mol), compared to the wild-type enzyme (15.5 ± 1.1 kJ/mol), confirming the contribution of Arg174 to the transition state formation of the catalytic reaction [48,49].

**Figure 7:**
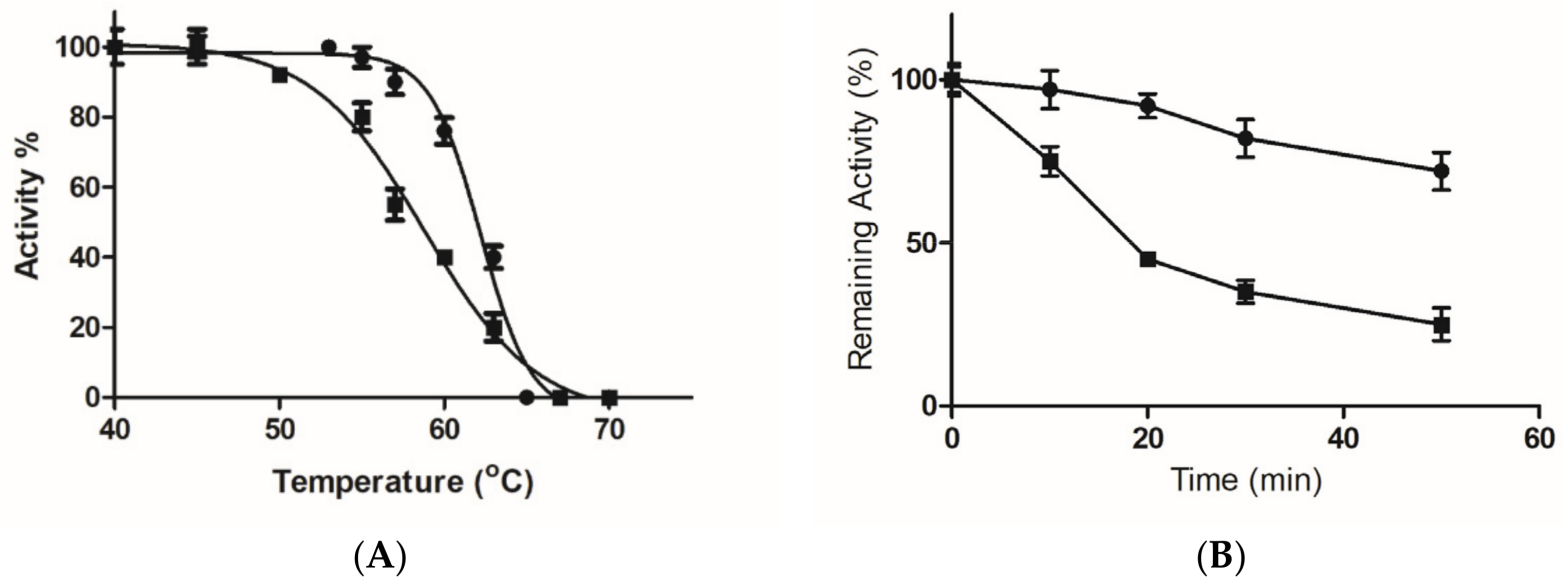
Effect of point mutation on thermal stability. (**A**). The residual activities of the wild-type enzyme (●) and the mutant enzyme Arg174Asn (■) were measured after heat treatment at various temperatures (°C). (**B**). Time course of thermal inactivation of the wild-type enzyme (●) and the mutant enzyme Arg174Asn (■) at 60 °C. At indicated times, enzyme samples were removed and assayed for residual FDH activity.

To assess whether the mutation of Arg174 had an effect on the structural stability of the *Cb*FDH, thermal inactivation studies were performed (Figure 7). The results showed that the mutant enzyme displays lower T_m_ value (58.7 ± 0.6 °C), compared to the wild-type enzyme (62.2 ± 0.4 °C) (Figure 7A). In addition, study of the time course of thermal inactivation at 60 °C showed that the wild-type and mutant enzyme retain about 72 and 20% of their initial activity, respectively, after 50 min (Figure 7B). Arg174 plays a central role in the formation of the nucleotide-binding site and is involved in a large number of interactions (Figure 2B). Previously published works have established that Arg residues in proteins contribute significantly to protein stability through the formation of strong electrostatic interactions [44,45]. In particular, the guanidinium group of Arg enables the formation of electrostatic interactions in three possible directions, which allows the development of a larger number of electrostatic interactions [44,45,46,47]. The relative high pK_a_ of the guanidinium group in Arg residues (~12.5) contributes to the formation of more stable and stronger electrostatic interactions, compared to that formed by Lys or His residues. Noteworthy, comparisons of the sequences of the thermophile-mesophile homologous protein pairs indicated that Arg is significantly more frequent in thermophilic proteins [50].

## 3. Materials and Methods

### 3.1. Materials

NAD^+^ (crystallised lithium salt, ca. 100%), NADH (disodium salt, grade II, 98%), ADP (disodium salt, 98%) and crystalline bovine serum albumin (fraction V) were obtained from Sigma-Aldrich (St. Louis, MO, USA). Molecular biology reagents were obtained from Invitrogen (Thermo Fisher Scientific, Waltham, MA, USA). The nucleotide analogue AMPS-HDB was synthesized as previously described by Vollmer et al., 1994 [38] for the 5′-*O*-[*S*-(4-bromo-2,3-dioxobutyl)]-thiophosphate. The replacement of the 4-bromo group by hydroxyl-group was achieved by incubation (5 h) in 0.03 M 2-(*N*-morpholino)ethanesulfonic acid (MES) buffer, pH 7.0 at 25 °C as previously described [38]. The structure was verified by electrospray tandem mass spectrometry (ESI/MS/MS) as described in [51].

### 3.2. Methods

#### 3.2.1. CbFDH Assays

Enzyme assays were performed at 25 °C according to a published method [5,52]. One unit of enzyme activity is defined as the amount that catalyses the conversion of 1 μmol of NAD^+^ to NADH and carbon dioxide per minute.

#### 3.2.2. Determination of Protein Concentration

Protein concentration was determined by the Bradford method using crystalline bovine serum albumin (fraction V) as standard [53].

#### 3.2.3. Expression and Purification of CbFDH

Recombinant *Cb*FDH was cloned and expressed in *E. coli* BL21(DE3) and purified by affinity chromatography on Cibacron Blue 3GA affinity column, as reported previously [5].

#### 3.2.4. Enzyme Inactivation and Inhibition Studies

Inactivation of the pure *Cb*FDH (1 U) by AMPS-HDB was carried out in experimental incubations that contained AMPS-HDB (0–0.5 mΜ) added in 50 mM borate buffer, pH 8.0. Identical control incubations in the absence of AMPS-HDB were also carried out. Aliquots (10–100 μL) were withdrawn at given time intervals and assayed for *Cb*FDH activity. Enzyme activity was plotted as log % of the initial (zero time) enzyme activity versus time in the experimental mixture. In protection studies, the control and experimental mixtures contained the same concentration of NAD^+^ (1 mM), NADH (1 mM), sodium formate (50 mM) and AMPS-HDB (0.1 mM). Inactivation rates were measured using the GraphPad Prism 5.0 software program. Inhibition studies with NADH (0.02–0.08) and AMP (2–10 mM) were conducted as described by Kato et al., 1979 [54], using NAD^+^ as a variable substrate (0.02 to 0.5 mM) in the presence of a saturation level of formate (50 mM).

#### 3.2.5. Site-Directed Mutagenesis

Site-directed mutagenesis was performed according to the unique site elimination method [55] as described elsewhere [52]. The oligonucleotide primer sequences that was used for the construction of the Arg174Asn mutation were: 5′-GGTGCCGGTAA*T*ATTGGTTACAGA-3′.

#### 3.2.6. Kinetic Analysis of Wild-Type and Mutant Enzymes

Steady-state kinetic measurements were performed in 0.1 M potassium phosphate buffer, pH 7.5 as described elsewhere [5,52]. All initial velocities were determined in triplicate. The kinetic parameters k_cat_ and K_m_ were calculated by non-linear regression analysis of experimental steady-state data using the GraphPad Prism 5.0 software program.

#### 3.2.7. Effect of Temperature on Enzyme Activity

The dependence of the enzyme activity on temperature was measured at different temperatures (10–60 °C) and analysed by the Arrhenius plot [48,49,56].

#### 3.2.8. Thermal Stability of the Wild-Type and Mutant Enzyme

Thermal inactivation of the wild-type and mutant Arg174Asn enzyme was monitored by activity measurements in 100 mM potassium phosphate buffer pH 7.4. Enzyme samples, were incubated (40 to 70 °C) for 20 min and following incubation, enzyme samples were assayed for residual FDH activity. The enzyme activity data were plotted as relative inactivation (%) versus temperature (°C). The T_m_ values (the temperature at which 50% of the initial enzyme activity is lost after heat treatment) were determined from such plots using the GraphPad Prism 5.0 software program. The time course of thermal inactivation of the wild-type and the mutant Arg174Asn was determined at 60 °C in 100 mM potassium phosphate buffer pH 7.4.

## 4. Conclusions

In conclusion, we have presented a combination of protein chemistry and protein engineering studies to characterise an important amino acid residue of *Cb*FDH that interacts with NAD^+^. AMPS-HDB is a dicarbonyl nucleotide analogue suitable for probing the reactivity and structural role of critical arginyl residues in nucleotide-binding enzymes. The interaction of AMPS-HDB with *Cb*FDH consistent with the two-step Kitz-Wilson model. Site-directed mutagenesis established that Arg174 is the target residue of AMPS-HDB. Kinetics analysis showed that Arg174 plays critical role in coenzyme binding and contributes to enzyme’s structural stability. The results of the present work also illustrate that AMPS-HDB can be used for assessing the functional and structural role of a particular Arg residue, located at the pyrophosphate-binding region of the Rossmann fold, in other enzymes and proteins.

## Figures and Tables

**Table 1:** Steady-state kinetic parameters for the wild-type and mutant Arg174Asn enzymes.

Enzyme	K_m_, (μM) (NAD^+^)	K_m_, (mM) (Formate)	k_cat_ (% of the Wild-Type)	K_i_ (mM) (NADH)	K_i_ (mM) (AMP)
Wild-type	0.04 ± 0.003	2.4 ± 0.1	100	0.03 ± 0.005	1.6 ± 0.1
Arg174Asn	1.27 ± 0.1	3.1 ± 0.2	17.5	1.1 ± 0.05	9.2 ± 0.3

## Data Availability

Data is contained within the article.

## Supplementary Materials

The following are available online, Figure S1: Comparison of the Circular Dichroism (CD) spectra of native and mutated enzyme.

## References

[1] Popov V.O., Lamzin V.S. (1994). NAD+-dependent formate dehydrogenase. Biochem. J..

[2] Bulut H., Valjakka J., Yuksel B., Yilmazer B., Turunen O., Binay B. (2020). Effect of Metal Ions on the Activity of Ten NAD-Dependent Formate Dehydrogenases. Protein J..

[3] Nielsen C.F., Lange L., Meyer A.S. (2019). Classification and enzyme kinetics of formate dehydrogenases for biomanufacturing via CO_2_ utilization. Biotechnol. Adv..

[4] Jormakka M., Byrne B., Iwata S. (2003). Formate dehydrogenase—A versatile enzyme in changing environments. Curr. Opin. Struct. Biol..

[5] Andreadeli A., Platis D., Tishkov V., Popov V., Labrou N.E. (2008). Structure-guided alteration of coenzyme specificity of formate dehydrogenase by saturation mutagenesis to enable efficient utilization of NADP+. FEBS J..

[6] Jiang H.-W., Chen Q., Pan J., Zheng G.-W., Xu J.-H. (2020). Rational Engineering of Formate Dehydrogenase Substrate/Cofactor Affinity for Better Performance in NADPH Regeneration. Appl. Biochem. Biotechnol..

[7] Guo X., Wang X., Liu Y., Li Q., Wang J., Liu W., Zhao Z.K. (2020). Structure-Guided Design of Formate Dehydrogenase for Regeneration of a Non-Natural Redox Cofactor. Chem.-A Eur. J..

[8] Luo W., Zhu J., Zhao Y., Zhang H., Yang X., Liu Y., Rao Z., Yu X. (2020). Cloning and Expression of a Novel Leucine Dehydrogenase: Characterization and L-tert-Leucine Production. Front. Bioeng. Biotechnol..

[9] Kim S., Lindner S.N., Aslan S., Yishai O., Wenk S., Schann K., Bar-Even A. (2020). Growth of E. coli on formate and methanol via the reductive glycine pathway. Nat. Chem. Biol..

[10] Guo X., Liu Y., Wang Q., Wang X., Li Q., Liu W., Zhao Z.K. (2019). Non-natural Cofactor and Formate-Driven Reductive Carboxylation of Pyruvate. Angew. Chem. Int. Ed..

[11] Agomuo E.N., Amadi P.U. (2020). Biochemical Implications of Biotransformation of Some Toxic Floras Using Natural Local Enzyme Sources. Recent Patents Biotechnol..

[12] Miyaji A., Amao Y. (2020). How does methylviologen cation radical supply two electrons to the formate dehydrogenase in the catalytic reduction process of CO_2_ to formate?. Phys. Chem. Chem. Phys..

[13] Tian Y., Zhou Y., Zong Y., Li J., Yang N., Zhang M., Guo Z., Song H. (2020). Construction of Functionally Compartmental Inorganic Photocatalyst–Enzyme System via Imitating Chloroplast for Efficient Photoreduction of CO_2_ to Formic Acid. ACS Appl. Mater. Interfaces.

[14] Calzadiaz-Ramirez L., Calvó-Tusell C., Stoffel G.M.M., Lindner S.N., Osuna S., Erb T.J., Garcia-Borràs M., Bar-Even A., Acevedo-Rocha C.G. (2020). In Vivo Selection for Formate Dehydrogenases with High Efficiency and Specificity toward NADP+. ACS Catal..

[15] Ren S., Wang Z., Bilal M., Feng Y., Jiang Y., Jia S., Cui J. (2020). Co-immobilization multienzyme nanoreactor with co-factor regeneration for conversion of CO2. Int. J. Biol. Macromol..

[16] Çakar M.M., Ruupunen J., Mangas-Sanchez J., Birmingham W.R., Yildirim D., Turunen O., Turner N.J., Valjakka J., Binay B. (2020). Engineered formate dehydrogenase from Chaetomium thermophilum, a promising enzymatic solution for biotechnical CO2 fixation. Biotechnol. Lett..

[17] Satanowski A., Bar-Even A. (2020). A one-carbon path for fixing CO_2_: C1 compounds, produced by chemical catalysis and upgraded via microbial fermentation, could become key intermediates in the valorization of CO2 into commodity chemicals. EMBO Rep..

[18] Yadav R.K., Baeg J.-O., Oh G.H., Park N.-J., Kong K.-J., Kim J., Hwang D.W., Biswas S.K. (2012). A Photocatalyst–Enzyme Coupled Artificial Photosynthesis System for Solar Energy in Production of Formic Acid from CO_2_. J. Am. Chem. Soc..

[19] Artiukhov A.V., Pometun A.A., Zubanova S.A., Tishkov V.I., Bunik V.I. (2020). Advantages of formate dehydrogenase reaction for efficient NAD+ quantification in biological samples. Anal. Biochem..

[20] Hovda K.E., Gadeholt G., Evtodienko V., Jacobsen D. (2015). A novel bedside diagnostic test for methanol poisoning using dry chemistry for formate. Scand. J. Clin. Lab. Investig..

[21] Hovda K.E., Urdal P., Jacobsen D. (2005). Increased Serum Formate in the Diagnosis of Methanol Poisoning. J. Anal. Toxicol..

[22] Pohanka M. (2019). Antidotes Against Methanol Poisoning: A Review. Mini-Rev. Med. Chem..

[23] Shin W.-H., Kihara D. (2019). 55 Years of the Rossmann Fold. Methods Mol. Biol..

[24] Hanukoglu I. (2015). Proteopedia: Rossmann fold: A beta-alpha-beta fold at dinucleotide binding sites. Biochem. Mol. Biol. Educ..

[25] Munsamy G., Soliman M.E.S. (2019). Unveiling a New Era in Malaria Therapeutics: A Tailored Molecular Approach Towards the Design of Plasmepsin IX Inhibitors. Protein J..

[26] Messaoudi A., Zoghlami M., Basharat Z., Sadfi-Zouaoui N. (2019). Identification of a Potential Inhibitor Targeting MurC Ligase of the Drug Resistant Pseudomonas aeruginosa Strain through Structure-Based Virtual Screening Approach and In Vitro Assay. Curr. Pharm. Biotechnol..

[27] Prathiviraj R., Chellapandi P. (2020). Deciphering Molecular Virulence Mechanism of Mycobacterium tuberculosis Dop isopeptidase Based on Its Sequence–Structure–Function Linkage. Protein J..

[28] Neetu N., Sharma M., Mahto J.K., Kumar P. (2020). Biophysical and In-Silico Studies of Phytochemicals Targeting Chorismate Synthase from Drug-Resistant Moraxella Catarrhalis. Protein J..

[29] Kataria R., Khatkar A. (2019). Molecular Docking of Natural Phenolic Compounds for the Screening of Urease Inhibitors. Curr. Pharm. Biotechnol..

[30] Mozafari M., Tariverdian T., Beynaghi A. (2020). Trends in Biotechnology at the Turn of the Millennium. Recent Patents Biotechnol..

[31] Dalmizrak O., Teralı K., Asuquo E.B., Ogus I.H., Ozer N. (2019). The Relevance of Glutathione Reductase Inhibition by Fluoxetine to Human Health and Disease: Insights Derived from a Combined Kinetic and Docking Study. Protein J..

[32] Bazaes S.E. (1996). Affinity labels as probes for the study of the nucleotide binding site of enzymes. Biol. Res..

[33] Labrou N.E., Muharram M.M., Abdelkader M.S. (2016). Delineation of the structural and functional role of Arg111 in GSTU4-4 from Glycine max by chemical modification and site-directed mutagenesis. Biochim. Biophys. Acta (BBA)-Proteins Proteom..

[34] Colman R.F., Huang Y.-C., King M.M., Erb M. (1984). 6-[(4-bromo-2,3-dioxobutyl)thio]-6-deaminoadenosine 5′-monophosphate and 5′-diphosphate: New affinity labels for purine nucleotide sites in proteins. Biochemistry.

[35] Chabot N., Vinatier V., Gefflaut T., Baudoin C., Rodriguez F., Blonski C., Hoffmann P. (2008). Irreversible inhibition of aldolase by a phosphorylated α-dicarbonyl compound. J. Enzym. Inhib. Med. Chem..

[36] Patthy L., Thész J. (1980). Origin of the selectivity of alpha-dicarbonyl reagents for arginyl residues of anion-binding sites. JBIC J. Biol. Inorg. Chem..

[37] Egorov A.M., Tishkov V.I., Popov V.O., Berezin I.V. (1981). Study of the role of arginine residues in bacterial formate dehydrogenase. Biochim. Biophys. Acta..

[38] Kayikci M., Venkatakrishnan A.J., Scott-Brown J., Ravarani C.N.J., Flock T., Babu M.M. (2018). Visualization and analysis of non-covalent contacts using the Protein Contacts Atlas. Nat. Struct. Mol. Biol..

[39] Vollmer S.H., Walner M.B., Tarbell K.V., Colman R.F. (1994). Guanosine 5′-0-[S-(4-Bromo-2,3-dioxobutyl)]thiophosphate and Adenosine 5′-0-[S-(4-Bromo-2,3- dioxobutyl)]thiophosphate: New Nucleotide Affinity Labels which React with Rabbit Muscle Pyruvate Kinase. J. Biol. Chem..

[40] Kitz R., Wilson I.B. (1962). Esters of Methanesulfonic Acid as Irreversible Inhibitors of Acetylcholinesterase. J. Biol. Chem..

[41] Kotzia G.A., Labrou N.E. (2004). S-(2,3-dichlorotriazinyl)glutathione. A new affinity label for probing the structure and function of glutathione transferases. Eur. J. Biochem..

[42] AlQarni M.H., Muharram M.M., Labrou N.E., AlQarni M.H. (2018). Ligand-induced glutathione transferase degradation as a therapeutic modality: Investigation of a new metal-mediated affinity cleavage strategy for human GSTP1-1. Int. J. Biol. Macromol..

[43] Platis M., Vlachakis D., Foudah A.I., Muharram M.M., AlQarni M.H., Papageorgiou A.C., Labrou N.E. (2020). The interaction of Schistosoma japonicum glutathione transferase with Cibacron blue 3GA and its fragments. Med. Chem..

[44] Sokalingam S., Raghunathan G., Soundrarajan N., Lee S.-G. (2012). A Study on the Effect of Surface Lysine to Arginine Mutagenesis on Protein Stability and Structure Using Green Fluorescent Protein. PLoS ONE.

[45] Kumar S., Tsai C.-J., Nussinov R. (2000). Factors enhancing protein thermostability. Protein Eng. Des. Sel..

[46] Marinou M., Platis D., Ataya F.S., Chronopoulou E., Vlachakis D., Labrou N.E. (2018). Structure-based design and application of a nucleotide coenzyme mimetic ligand: Application to the affinity purification of nucleotide dependent enzymes. J. Chromatogr. A.

[47] Patel N., Shahane S., Majumdar R., Mishra U. (2019). Mode of Action, Properties, Production, and Application of Laccase: A Review. Recent Patents Biotechnol..

[48] Fan Y.-X., McPHIE P., Miles E.W. (2000). Regulation of Tryptophan Synthase by Temperature, Monovalent Cations, and an Allosteric Ligand. Evidence from Arrhenius Plots, Absorption Spectra, and Primary Kinetic Isotope Effects. Biochemistry.

[49] Peng G., Fritzsch G., Zickermann V., Schagger H., Mentele R., Lottspeich F., Bostina M., Radermacher M., Huber R., Stetter K.O. (2003). Isolation, Characterization and Electron Microscopic Single Particle Analysis of the NADH:Ubiquinone Oxidoreductase (Complex I) from the Hyperthermophilic Eubacterium Aquifex aeolicus. Biochemistry.

[50] Zeeshan F., Tabbassum M., Kesharwani P. (2019). Investigation on Secondary Structure Alterations of Protein Drugs as an Indicator of Their Biological Activity Upon Thermal Exposure. Protein J..

[51] Strzelecka D., Chmielinski S., Bednarek S., Jemielity J., Kowalska J. (2017). Analysis of mononucleotides by tandem mass spectrometry: Investigation of fragmentation pathways for phosphate- and ribose-modified nucleotide analogues. Sci. Rep..

[52] Labrou N.E., Rigden D.J. (2001). Active-site characterization of Candida boidinii formate dehydrogenase. Biochem. J..

[53] Bradford M.A. (1976). A rapid and sensitive method for the quantitation of microgram quantities of protein utilizing the principle of protein-dye binding. Anal. Biochem..

[54] Kato N., Sahm H., Wagner F. (1979). Steady-state kinetics of formaldehyde dehydrogenase and formate dehydrogenase from a methanol-utilizing yeast, Candida boidinii. Biochim. Biophys. Acta (BBA)-Enzym..

[55] Deng W.P., Nickoloff J.A. (1992). Site-directed mutagenesis of virtually any plasmid by eliminating a unique site. Anal. Biochem..

[56] Labrou N.E., Kotzia G.A., Clonis Y.D. (2004). Engineering the xenobiotic substrate specificity of maize glutathione S-transferase I. Protein Eng. Des. Sel..

