# Supplementary material for: Probing the Role of the Conserved Arg174 in Formate Dehydrogenase by Chemical Modification and Site-Directed Mutagenesis
Source: Molecules. 2021 Feb 25;26(5):1222. doi: 10.3390/molecules26051222 (PMC7956174; doi:10.3390/molecules26051222)
Supplement: Supplementary file 1 [file molecules-26-01222-s001.pdf]

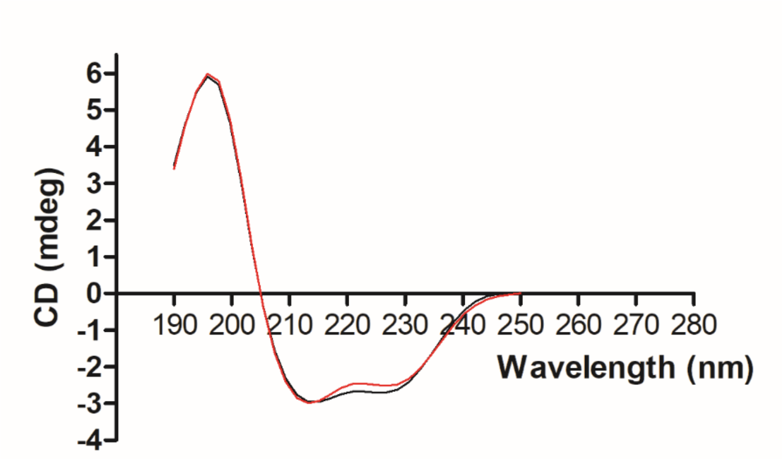

Supplementary Fig. 1.

CD measurements were conducted (Jasco J-1500) between 190 nm and 250 nm at 25 °C. The wild-type (red line) and mutant enzyme (black line) were diluted to 0.25 mg/mL using the sodium phosphate buffer (pH 7.4).
